# Kentucky

**Published:** 1897-08

**Authors:** 


*Kentucky.* An Act has been passed in this State providing for the destruction of horses suffering with glanders or farcy. Owners of animals destroyed will be reimbursed to the extent of fifty dollars per head.

